# *Neisseria meningitidis* Infecting a Prosthetic Knee Joint: A New Case of an Unusual Disease

**DOI:** 10.1155/2017/4545721

**Published:** 2017-02-23

**Authors:** Berta Becerril Carral, Elvira Alarcón Manoja, Salvador López Cárdenas, Jesús Canueto Quintero

**Affiliations:** ^1^Unidad Clínica de Gestión de Enfermedades Infecciosas y Microbiología del Área Sanitaria del Campo de Gibraltar, Cádiz, Spain; ^2^Unidad Clínica de Gestión de Medicina Interna del Área Sanitaria del Campo de Gibraltar, Cádiz, Spain

## Abstract

Primary meningococcal meningitis is an infrequent but known disease. However, the infection of a prosthetic joint with* Neisseria meningitidis *is rare. We hereby describe the second case of an arthroplasty infected with* Neisseria meningitidis *that responded favourably to prosthesis retention with surgical debridement, in combination with antibiotics treatment.

## 1. Introduction

*Neisseria meningitidis *is a Gram-negative, facultative, aerobic, or anaerobic diplococcus. It was isolated for the first time by Anton Weischselbaum in 1887 in the cerebrospinal fluid of a meningitis patient [1]. It is a strict human pathogen with a wide variety of clinical manifestations.* N. meningitidis *can cause extremely severe diseases, such as meningitis and life-threatening sepsis, as well as the asymptomatic colonization of the nasopharynx in approximately 10% of the population during endemic periods of infection [2]. It rarely provokes localized forms, such as septic arthritis, pneumonia, pericarditis, otitis, sinusitis, and urethritis [3]. Primary purulent arthritis caused by* N. meningitidis *is a rare but known disease. However, as far as we know, there is only one published case report of an arthroplasty infected with* Neisseria meningitidis* [4]. We hereby present a new case of this exceptional infection.

The duration and form of administration of antibiotic agents in these infections are based on expert opinions. The traditional recommendation duration of total postdebridement antibiotic treatment of 6 to maximal 12 weeks or the duration of its initial parental part (2–4 weeks) is discussed in favour of an oral antibiotic treatment from the start [5].

## 2. Case Presentation

A 78-year-old Caucasian female patient with dislipemia, stable ischemic cardiopathology, degenerative aortic and mitral valves, and generalized osteoarthritis arrived at the Emergency Department of Hospital Punta de Europa, Algeciras, Spain, a 328-bed general hospital, with a chief complaint of pain in her left knee. The patient had undergone a total left knee arthroplasty 7 months before due to a severe functional limitation secondary to osteoarthritis. Seven days before admission and while experiencing symptoms of pharyngitis, she had an accident at home and suffered a sprain in her left ankle. The patient complained of pain and swelling in the above joint that radiated towards her left knee. Within days of the accident, the patient reported a gradual reduction in the symptoms in her ankle but an increase of pain in her knee. She started complaining of intense pain, functional impairment, redness, and increase of local temperature but without evidence of skin rash. The presence of intra-articular fluid was confirmed in the operated knee. There were no other affected joints or presence of skin lesions. Main analytical data were as follows: leukocytes: 13.6 × 109/L with 80% granulocytes; haemoglobin: 11.8 g/dL; platelets: 486 × 109/L; immunoglobulin levels in serum were normal. An arthrocentesis was performed and it yielded an amber-coloured fluid with 9.9 × 109/L (67% polymorphonuclear), glucose: 97 mg/dL, proteins: 4.2 g/dL, and LDH: 1866 U/L. No crystals were observed. The joint fluid culture was positive for* N. meningitidis* serogroup B (latex agglutination (Difco™ Neisseria Meningitidis Antiserum, Becton, Dickinson and Company®, Sparks, MD)), with an intermediate sensitivity to penicillin (minimum inhibitory concentration (MIC): 0,25 microg/ml by *E*-test (BioMerieux® Inc, Marcy L'Etoil, France)) and sensitivity to cefotaxime (0,01 microg/ml by *E*-test (BioMerieux Inc, Marcy L'Etoil, France)) and ciprofloxacin (by disk diffusion (Sensi-Disc™, Becton, Dickinson and Company, Sparks, MD) according to the CLSI guidelines [6, 7].* N. meningitidis* was identified by growth on chocolate agar and blood agar, and by utilization of enriched media (API NH biochemical testing (BioMerieux Inc, Marcy L'Etoil, France)). Blood cultures were negatives (BacT/ALERT® 3D system (BioMerieux Inc, Mercy L'Etoil, France)). The procedure included arthroscopic debridement with implant retention because of individual patient circumstances and acute onset of symptoms in the setting of a well-fixed prosthesis without a sinus tract and antibiotics and 2 grams ceftriaxone administered intravenously every 24 hours over a 3-week period, followed by oral administration of 750 mg ciprofloxacin every 12 hours over the total 12-week study period. No recurrence has been observed during a two-year follow-up. The Department of Preventive Medicine and Public Health was notified of the case and the recommendations on prophylaxis and treatment of possible carriers were followed, and the patient was vaccinated with meningococcal vaccines following this episode [8].

## 3. Discussion

Infection is the most feared complication after an arthroplasty and has proven to be the cause for a notable rate of morbidity and a nonnegligible rate of mortality [9]. There are two ways of how microorganisms can infect a prosthetic joint, and the most frequent is direct inoculation during surgery. The second one is haematogenous dissemination, responsible for 10% of the cases.

In his article, Aslam et al. [10] include nonsurgical trauma to the implant as a risk factor for the acquisition of a haematogenous prosthetic infection. In the case described, a traumatic event that had happened in the past and that had affected the left ankle and knee could have created an environment allowing the bacteria, which just happened to be in the blood at the time due to a concomitant pharyngitis, to seed the prosthetic joint.

The most frequently isolated bacteria in prosthetic joint infections are:* Staphylococcus aureus*,* Streptococcus *spp., and Gram-negative bacilli, although the proportions vary depending on the series [11].

Three clinical syndromes have been described in association to the bloodstream infection with* Neisseria meningitidis*. The first one, acute meningococcemia, is a severe condition that occurs within the context of a sepsis, a septic shock, or meningitis. Although rarer, bacteraemia adopts a less aggressive clinical course, such as chronic meningococcemia, indistinguishable from chronic gonococcemia, which is a rare infection characterized by intermittent fever episodes, maculopapular rash, and arthralgia; it can also adopt the form of transient meningococcemia, characterized by fever and unspecified rash over a 2–5-day evolution period where presence of* N. meningitidis *in the blood usually comes as an unexpected finding [12].

Besides, the ability of* N. meningitidis *to infect prosthetic joints shall depend upon the affinity for the osteoarticular tissue, the type of prosthesis employed, and the residual joint tissue. The frequency of arthritis complicating acute meningococcal disease in adults ranges from 4 to 50%. Schaad [13] describes the following three clinical types of arthritis in meningococcal disease. (1) The most common type is arthritis complicating acute meningococcal disease. Its mechanism is of an immunologic basis and affects larger joints, mainly knees, and, less frequently, the direct invasion of the joint by the bacteria. (2) Chronic meningococcemia is accompanied more often by arthralgia. (3) Primary meningococcal arthritis, which is a rare form of acute septic arthritis, affects large joints almost exclusively and is monoarticular in two-thirds of the cases.

Both the case described by Vikram et al. [4] and the present description should be classified in the third category, where the germ's route of entry is supposedly haematogenous.

Biofilm formation plays a crucial role in the ability of germs to colonize nonbiological surfaces. Both encapsulated and nonencapsulated forms of* N. meningitidis* are capable of producing biofilm. This favours its persistence in the nasopharynx and seems to play an important role in the process of colonization and the carrier status (in this case the pharyngitis seven days before admission was the probable entry site but was not confirmed). Nonetheless, the role of biofilm-forming strains in the different clinical syndromes associated with the meningococcal disease is still to be determined.

To our knowledge, this is the second reported case of primary meningococcal arthritis in a prosthetic joint. The previous case, published in 2001, described the infection of a total knee prosthesis by* N. meningitidis *serogroup Y in an 80-year-old female patient. This patient's prosthesis had been placed 3 years before and, as in the case hereby presented, it did not have any distinctive clinical feature. The management included prosthesis retention and a 6-week course of i.v. ceftriaxone.

In both cases, the most likely route of entry for the germ into the joint was the haematogenous route, although the presence of this germ in blood could never be confirmed. The case presented was probably favoured by a minor local trauma. Besides, the infection of other types of nonarticular prosthetic grafts with* N. meningitidis *is very rare and limited to a very reduced number of endocarditis on prosthetic valves [14, 15]. Therefore,* N. meningitidis *is a germ that has low affinity for the joint tissue and, although capable of producing biofilm in order to survive in the surface secretions of the nasopharynx, it has very limited capacity to cause infections in abiotic surfaces, and here lies the exceptional significance of the case hereby presented.
